# Supplementary material for: PIBF1 regulates trophoblast syncytialization and promotes cardiovascular development
Source: Nat Commun. 2024 Feb 19;15:1487. doi: 10.1038/s41467-024-45647-8 (PMC10876648; doi:10.1038/s41467-024-45647-8)
Supplement: Supplementary file 1 — Supplementary Information [file 41467_2024_45647_MOESM1_ESM.pdf]

Supplementary information for

PIBF1 regulates trophoblast syncytialization and promotes cardiovascular development

Jong Geol Lee[1,2,19], Jung-Min Yon[1,3,19], Globinna Kim[1,3], Seul-Gi Lee[4], C-Yoon Kim[5], Seung-A Cheong[1], Hyun-Yi Kim[6], Jiyoung Yu[1], Kyunggon Kim[1,7], Young Hoon Sung[1,3], Hyun Ju Yoo[1,7], Dong-Cheol Woo[1,8], Jin Kyung Rho[1,9], Chang Hoon Ha[1,9], Chan-Gi Pack[1,8], Seak Hee Oh[10], Joon Seo Lim[1], Yu Mi Han[11], Eui-Ju Hong[12], Je Kyung Seong[2,13], Han-Woong Lee[14], Sang-Wook Lee[2,15], Ki-Up Lee[1,9], Chong Jai Kim[1,16], Sang-Yoon Nam[17], You Sook Cho[1,18]*, In-Jeong Baek[1,2,3]*

Affiliations:

[1]Asan Institute for Life Sciences, Asan Medical Center; Seoul, 05505, Korea.

[2]Korea Mouse Phenotyping Center (KMPC); Seoul, 08826, Korea.

[3]Department of Cell and Genetic Engineering, Asan Medical Center, University of Ulsan College of Medicine; Seoul, 05505, Korea.

[4]Department of Stem Cell Biology, School of Medicine, Konkuk University; Seoul, 05029, Korea.

[5]College of Veterinary Medicine, Konkuk University; Seoul, 05029, Korea.

[6]NGeneS Inc.; Ansan, 15495, Korea.

[7]Department of Digital Medicine, Asan Medical Center, University of Ulsan College of Medicine; Seoul, 05505, Korea.

[8]Department of Biomedical Engineering, Asan Medical Center, University of Ulsan College of Medicine; Seoul, 05505, Korea.

[9]Department of Biochemistry and Molecular Biology, Asan Medical Center, University of Ulsan College of Medicine; Seoul, 05505, Korea.

[10]Department of Pediatrics, Asan Medical Center, University of Ulsan College of Medicine; Seoul, 05505, Korea.

[11]Research Institute of Medical Science, Sungkyunkwan University School of Medicine, Seoul, 06351, Korea.

[12]College of Veterinary Medicine, Chungnam National University; Daejeon, 34134, Korea.

[13]College of Veterinary Medicine, Seoul National University; Seoul, 08826, Korea.

[14]Department of Biochemistry, College of Life Science and Biotechnology, Yonsei University; Seoul, 03722, Korea.

[15]Department of Radiation Oncology, Asan Medical Center, University of Ulsan College of Medicine; Seoul, 05505, Korea.

[16]Department of Pathology, Asan Medical Center, University of Ulsan College of Medicine; Seoul, 05505, Korea.

[17]College of Veterinary Medicine, Chungbuk National University; Cheongju, 28644, Korea.

[18]Division of Allergy and Clinical Immunology, Department of Internal Medicine, Asan Medical Center, University of Ulsan College of Medicine; Seoul, 05505, Korea.

[19]These authors contributed equally to this work: Jong Geol Lee, Jung-Min Yon

*Corresponding authors. Email: ijbaek@amc.seou.kr, yscho@amc.seoul.kr

The PDF file includes:

Supplementary Figures (Fig. S#)

Fig. S1. Schematic diagram of two crRNAs targeting exon 2 or 4 of human *PIBF1*.

Fig. S2. Trophoblast syncytialization is disrupted in *PIBF1* KO BeWo cells.

Fig. S3. *PIBF1* KO leads to a declined differentiation features of TSC with its self-renewal capacity scarcely affected.

Fig. S4. CRISPR/Cpf1-generated *PIBF1* KO hTSCs exhibit defects in differentiation into EVT and SynT.

Fig. S5. Exogenous treatment of recombinant PIBF1 (rPIBF) has no impact on the decreased syncytial formation of *PIBF1* KO BeWo cells.

Fig. S6. PIBF1 coordinates vasculature formation by modulating the recruitment and angiogenesis of ECs and placental pericytes (Pl-PC) *in vitro*.

Fig. S7. *Pibf1*-null mice display *in utero* lethality, and its time window of impaired embryogenesis is associated with the primary placentation period.

Fig. S8. Viability of *Pibf1*-null embryos is not associated with the maternal genetic dose of *Pibf1* or uterine immune tolerance regarding natural killer (uNK) cells/lymphocytes.

Fig. S9. *In situ* hybridization (ISH) and immunohistochemistry (IHC) assays of Pibf1 in the mouse placenta and embryo at E10.5.

Fig. S10. Epiblast-specific *Pibf1* knockout using a *Meox2*-driven cre line.

Fig. S11. *Meox2*-, *Cdh5*- or *Tie2*-driven cre recombinase activity in the placenta and the embryonic heart.

Fig. S12. Supporting data for defective developing hematopoietic tissues of *Pibf1*-null conceptus.

Fig. S13. Supporting data for *Pibf1*$^{\Delta/\Delta}$;*Meox2*$^{cre/+}$ placenta phenotype.

Fig. S14. *Pibf1*-null embryonic lethality is not *Trp53*-dependent, and ciliopathy-related anomalies are seen in *Pibf1*$^{\Delta/\Delta}$;*Meox2*$^{cre/+}$ fetuses at late gestation.

Fig. S15. Loss of *Pibf1* in hematopoietic lineages does not disrupt normal hematopoiesis.

Fig. S16. Deficiency of *Pibf1* in maternal blood and immune cells does not disrupt normal fertility.

Fig. S17. Dose effect of sPIBF1 on hHO development.

Fig. S18. Volcano plot of all genes expressed in rsPIBF-treated human heart organoids.

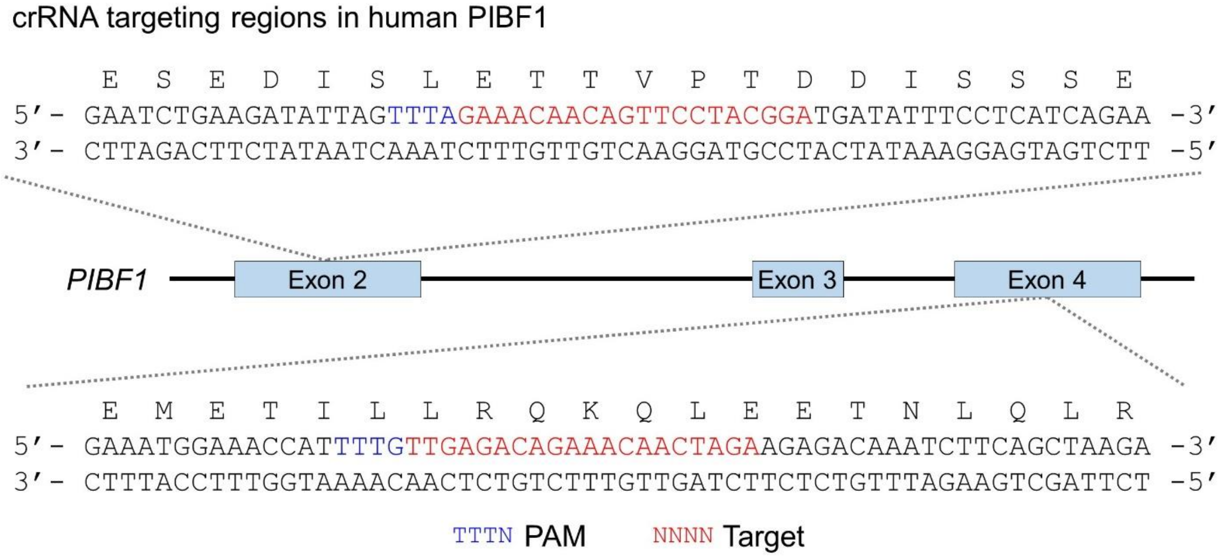

Supplementary Fig. 1. Schematic diagram of two crRNAs targeting exon 2 or 4 of human *PIBF1*.

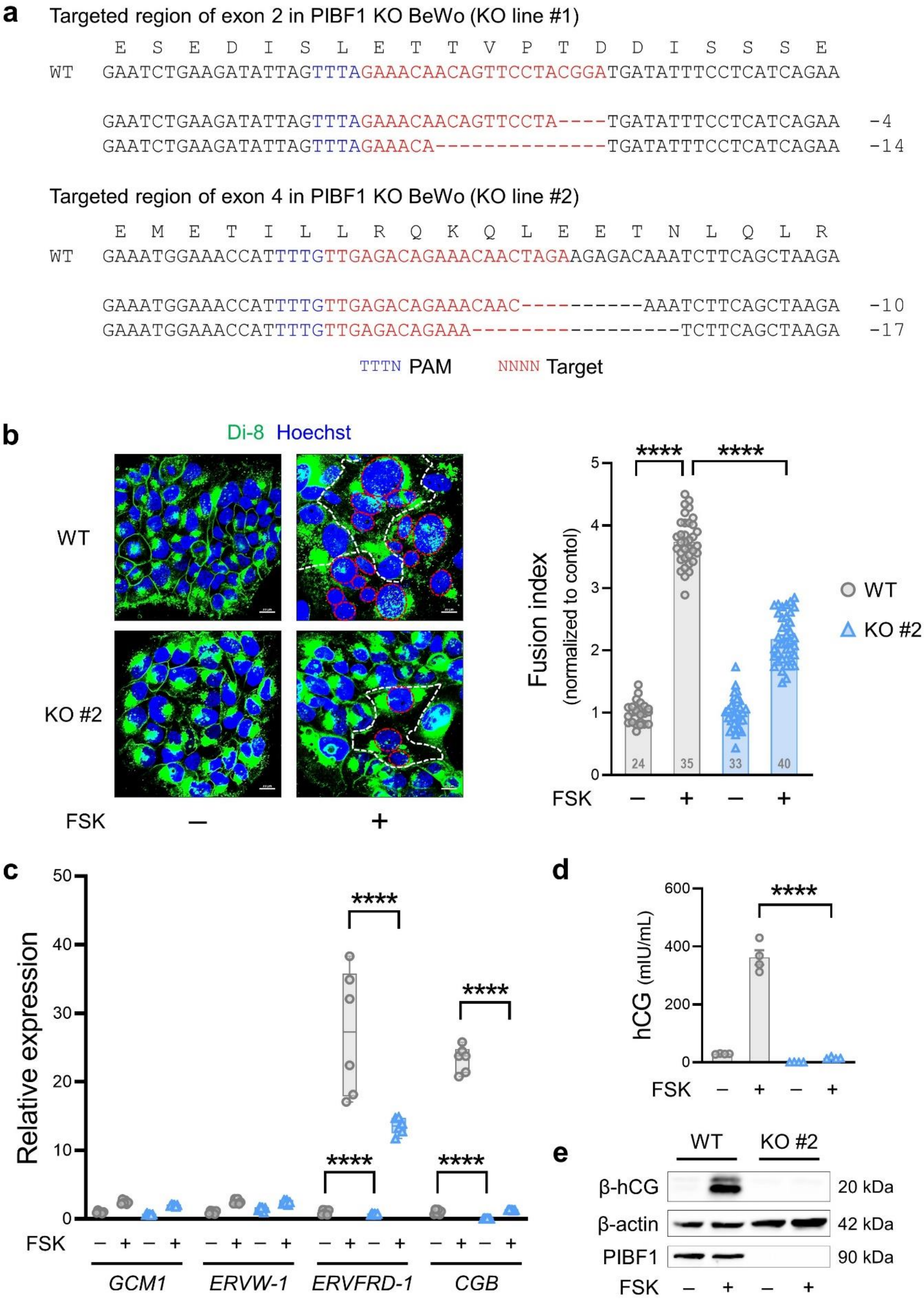

a
Targeted region of exon 2 in PIBF1 KO BeWo (KO line #1)
E S E D I S L E T T V P T D D I S S S E
WT GAATCTGAAGATATTAGTTTAGAAACAACAGTTCCTACGGATGATATTTCCTCATCAGAA
GAATCTGAAGATATTAGTTTAGAAACAACAGTTCCTA----TGATATTTCCTCATCAGAA -4
GAATCTGAAGATATTAGTTTAGAAACA--------------TGATATTTCCTCATCAGAA -14
Targeted region of exon 4 in PIBF1 KO BeWo (KO line #2)
E M E T I L L R Q K Q L E E T N L Q L R
WT GAAATGGAAACCATTTTGTTGAGACAGAAACAACTAGAAGAGACAAATCTTCAGCTAAGA
GAAATGGAAACCATTTTGTTGAGACAGAAACAAC----------AAATCTTCAGCTAAGA -10
GAAATGGAAACCATTTTGTTGAGACAGAAA-----------------TCTTCAGCTAAGA -17
TTTN PAM
NNNN Target
b
Di-8 Hoechst
WT
KO #2
FSK – +
Fusion index (normalized to contol)
**** ****
WT
KO #2
24 35 33 40
FSK – + – +
c
Relative expression
****
****
**** ****
FSK – + – + – + – + – + – + – + – +
GCM1 ERVW-1 ERVFRD-1 CGB
d
hCG (mIU/mL)
****
FSK – + – +
e
WT KO #2
β-hCG 20 kDa
β-actin 42 kDa
PIBF1 90 kDa
FSK – + – +

Supplementary Fig. 2. Trophoblast syncytialization is disrupted in *PIBF1* KO BeWo cells.
a Mutant *PIBF1* sequence observed in the *PIBF1* KO BeWo cell lines #1 and #2 using CRISPR/Cpf1 targeting human PIBF1 exon 2 and 4, respectively. **b** Trophoblast fusion assay in WT and BeWo *PIBF1* KO cell line #2. BeWo boundaries and nuclei were stained with Di-8-ANEPPS (Di-8; green) and Hoechst (blue). White and red dotted lines delineate the plasma membrane and nuclei within the fused cells. Data are expressed relative to that of WT control. **c** Expressions of SynT markers in FSK-treated *PIBF1* KO BeWo cell line #2. Data were normalized using *RPS18* and expressed relative to that of WT. **d** Levels of hCG secreted by FSK-treated *PIBF1* KO BeWo cell line #2. **e** Expressions of β-hCG and PIBF1 in FSK-treated *PIBF1* KO BeWo cell line #2. β-actin was used as an internal control. Data are presented as mean ± SEM (**b**, **d**) or mean ± minimum to maximum (**c**); ****$P < 0.0001$ in one-way ANOVA. Source data are provided as a Source Data file.

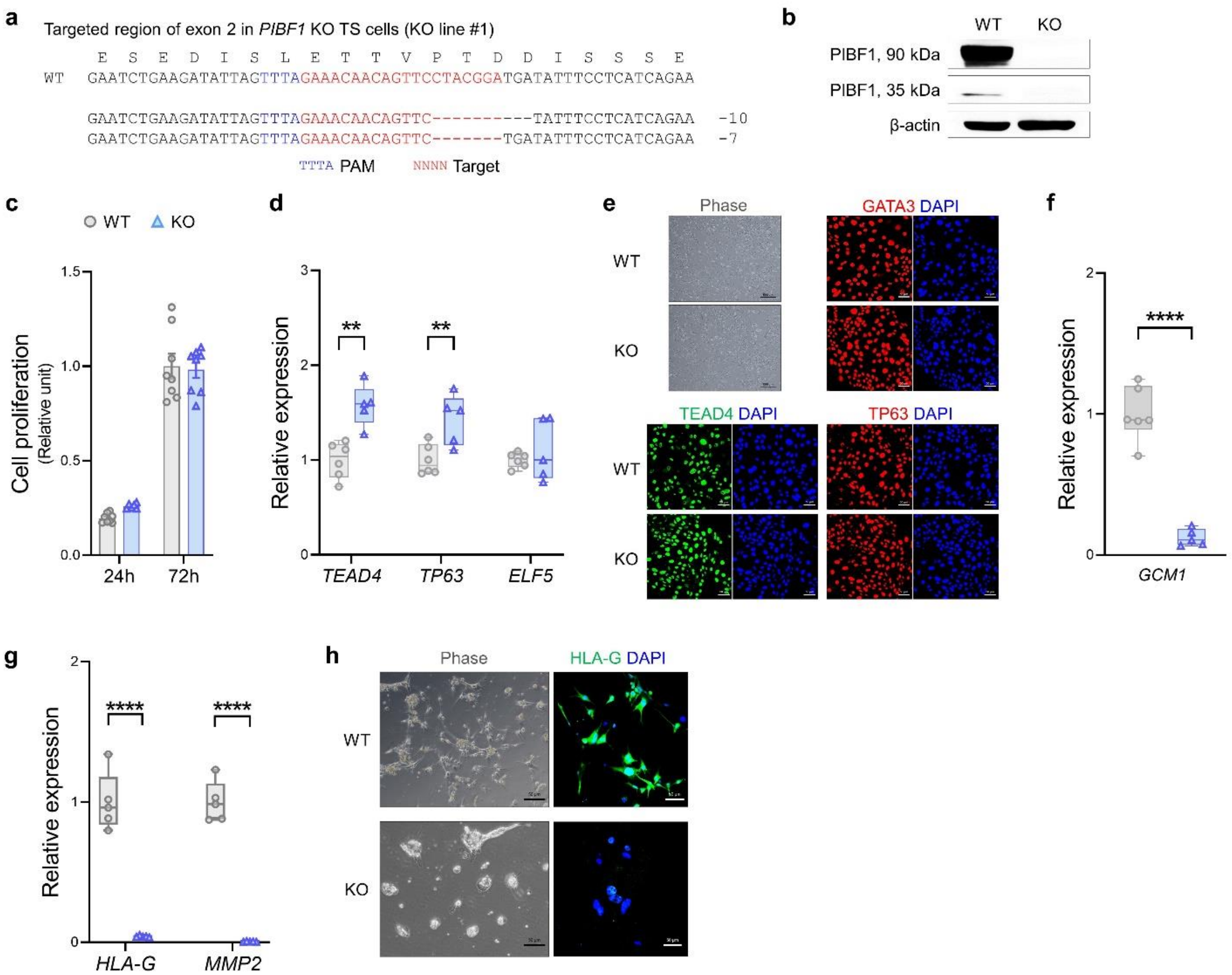

Supplementary Fig. 3. *PIBF1* KO leads to a declined differentiation features of TSC with its self-renewal capacity scarcely affected.

a Mutant *PIBF1* sequence observed in the *PIBF1* KO TSC using CRISPR/Cpf1 targeting human *PIBF1* exon 2. **b** Expression of PIBF1 in *PIBF1* KO TSC. β-actin was used as an internal control. **c** Cell proliferation rate in *PIBF1* KO TSC in a stem cell condition. Data are expressed relative to that of WT at 72 h and presented as mean ± SEM. **d, e** Expressions of TS markers at transcript (**d**) and protein levels (**e**) in *PIBF1* KO TSC in a stem cell condition. **f** Expression of *GCM1* transcript in an undifferentiated *PIBF1* KO TSC. **g** Expressions of *HLA-G* and *MMP2* in *PIBF1* KO TSC derived EVT (TS-EVT). **h** Phase contrast and HLA-G (green)-immunostained images in *PIBF1* KO TS-EVT. Transcript expression data (**d**, **f**, and **g**) were normalized using *GAPDH*, expressed relative to that of WT, and presented as mean ± minimum to maximum; $^{**}P < 0.01$; $^{****}P < 0.0001$ in Student's t-test. Source data are provided as a Source Data file.

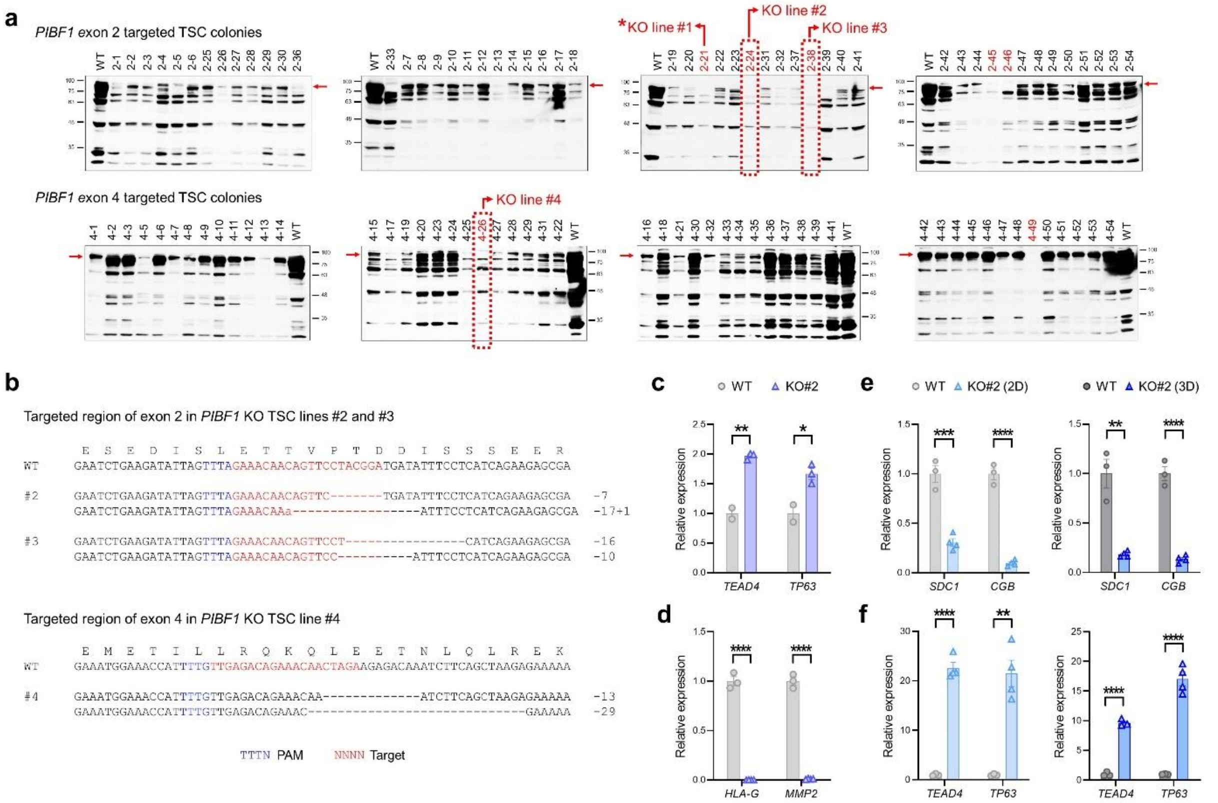

Supplementary Fig. 4. CRISPR/Cpf1-generated *PIBF1* KO hTSCs exhibit defects in differentiation into EVT and SynT.

a PIBF1 expression in *PIBF1* KO candidate hTSC colonies. Red arrows indicate a band size of full-length PIBF1 (90 kDa). Suspected colonies are colored in red. Colonies marked with a red dotted box were further analyzed using Sanger sequencing. Note that the data regarding TS KO lines #2-21 were described as KO line #1 in Figs. 1 and 2, and Supplementary Fig. 3. **b** Mutant *PIBF1* sequence observed in the *PIBF1* KO hTSC lines #2 to #4 using CRISPR/Cpf1 targeting human *PIBF1* exon 2 or 4. **c** Expressions of TS markers in *PIBF1* KO hTSC line #2 in a stem cell condition. **d** Expressions of *HLA-G* and *MMP2* in EVT derived from *PIBF1* KO hTSC line #2. **e, f** Expressions of SynT (**e**) and TS (**f**) markers in SynT derived from *PIBF1* KO hTSC line #2 in a 2D and 3D culture. Transcript expression data were normalized to *GAPDH* and expressed relative to WT (**c**-**f**). Data are presented as mean ± SEM (**c**-**f**); *$P < 0.05$; **$P < 0.01$; ***$P < 0.001$; ****$P < 0.0001$ in Student's t-test (**c**-**f**). Source data are provided as a Source Data file.

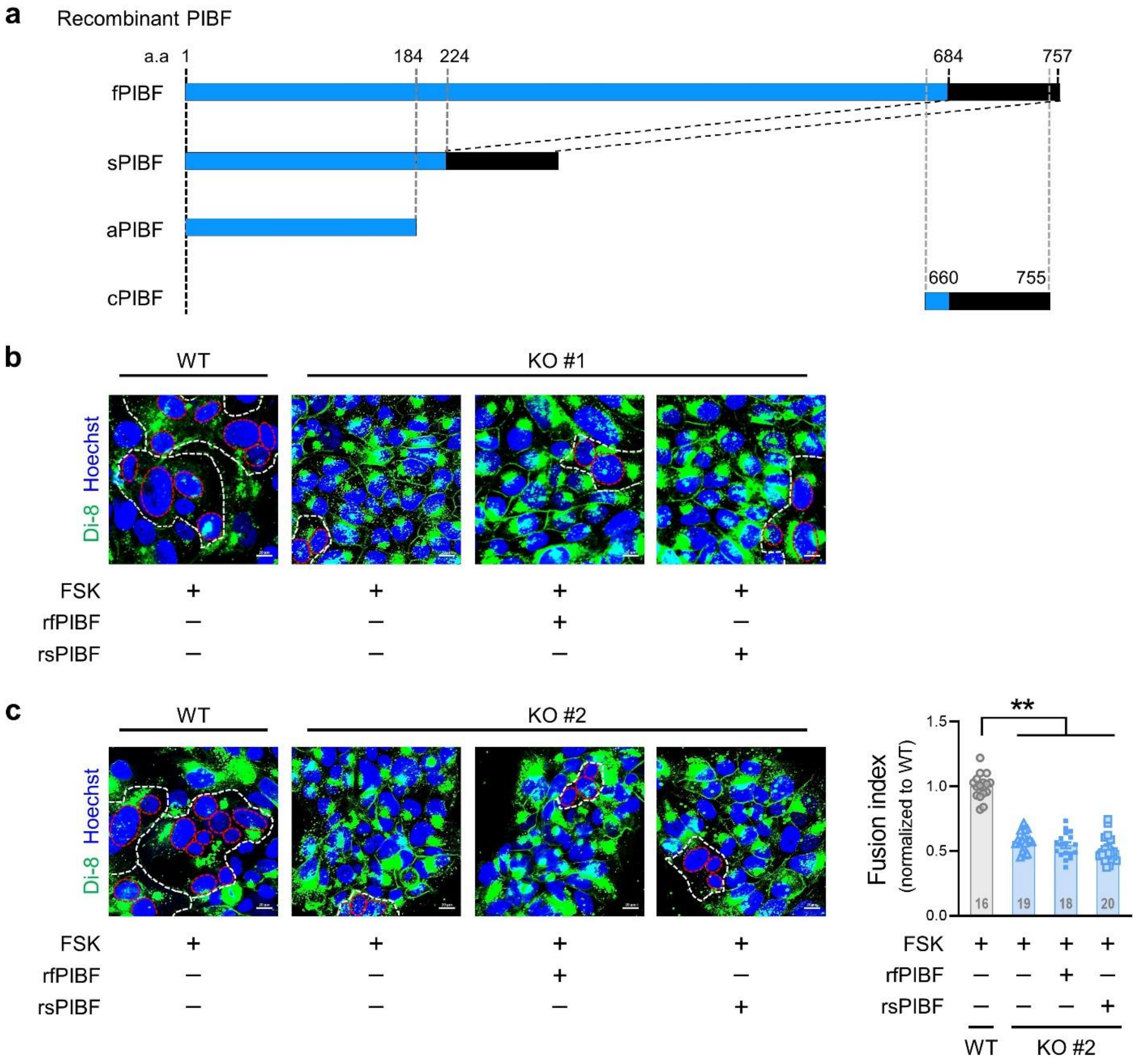

Supplementary Fig. 5. Exogenous treatment of recombinant PIBF1 (rPIBF) has no impact on the decreased syncytial formation of *PIBF1* KO BeWo cells.

a Schematic diagram of rPIBF used in these studies, including full length (fPIBF), secretory form (sPIBF), active domain (aPIBF), and C-terminal fragment of PIBF1 (cPIBF). **b** Representative images of recombinant sPIBF (rsPIBF) or rfPIBF-treated *PIBF1* KO BeWo cell line #1. **c** Fusion assay in *PIBF1* KO BeWo cell line #2 supplemented with rsPIBF or rfPIBF. Data are expressed relative to that of WT, and presented as mean ± SEM. ***P*<0.01 in one-way ANOVA. Source data are provided as a Source Data file.

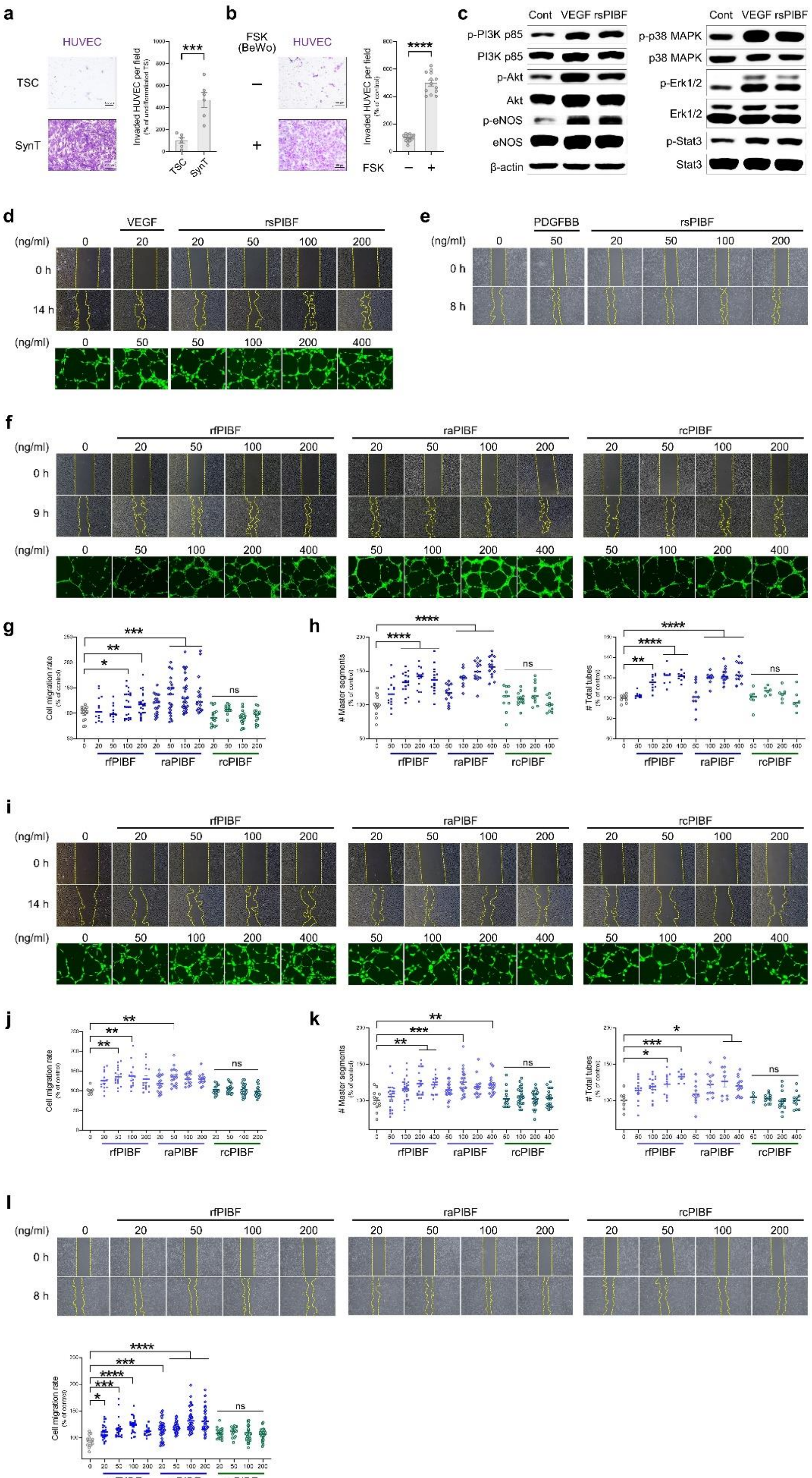
a
HUVEC
TSC
SynT
Invaded HUVEC per field
***
TSC
SynT
b
FSK (BeWo)
HUVEC
–
+
Invaded HUVEC per field
****
FSK – +
c
Cont VEGF rsPIBF
p-PI3K p85
PI3K p85
p-Akt
Akt
p-eNOS
eNOS
β-actin
Cont VEGF rsPIBF
p-p38 MAPK
p38 MAPK
p-Erk1/2
Erk1/2
p-Stat3
Stat3
d
VEGF
rsPIBF
(ng/ml) 0 20 20 50 100 200
0 h
14 h
(ng/ml) 0 50 50 100 200 400
e
PDGFBB
rsPIBF
(ng/ml) 0 50 20 50 100 200
0 h
8 h
f
rfPIBF
raPIBF
rcPIBF
(ng/ml) 0 20 50 100 200 20 50 100 200 20 50 100 200
0 h
9 h
(ng/ml) 0 50 100 200 400 50 100 200 400 50 100 200 400
g
Cell migration rate
***
**
*
ns
rfPIBF raPIBF rcPIBF
h
Master segments
****
****
ns
rfPIBF raPIBF rcPIBF
Total tubes
****
****
**
ns
rfPIBF raPIBF rcPIBF
i
rfPIBF
raPIBF
rcPIBF
(ng/ml) 0 20 50 100 200 20 50 100 200 20 50 100 200
0 h
14 h
(ng/ml) 0 50 100 200 400 50 100 200 400 50 100 200 400
j
Cell migration rate
**
**
**
ns
rfPIBF raPIBF rcPIBF
k
Master segments
**
***
**
ns
rfPIBF raPIBF rcPIBF
Total tubes
*
***
*
ns
rfPIBF raPIBF rcPIBF
l
rfPIBF
raPIBF
rcPIBF
(ng/ml) 0 20 50 100 200 20 50 100 200 20 50 100 200
0 h
8 h
Cell migration rate
****
***
****
***
*
ns
rfPIBF raPIBF rcPIBF

Supplementary Fig. 6. PIBF1 coordinates vasculature formation by modulating the recruitment and angiogenesis of ECs and placental pericytes (Pl-PC) *in vitro*.

a, b Recruitment of HUVEC by hTSC-derived SynT (**a**) and syncytialized BeWo cells (**b**) in a transwell invasion assay. Data are expressed relative to the control and presented as mean ± SEM. ***P<0.001, ****P<0.0001 in Student's t-test. **c** Representative blot images of angiogenesis-related signaling cascades in rsPIBF-treated HUVEC. β-actin was used as an internal control. **d, e** Representative images of migration and tube forming assay in rsPIBF-treated HUAEC (**d**) and migration assay in rsPIBF-treated Pl-PC (**e**). **f-h** Representative images of migration and tube forming assay in rfPIBF, raPIBF, or rcPIBF-treated HUVEC (**f**) and quantified results of their migration (**g**) and tube forming assay (**h**). **i-k** Representative images of migration and tube forming assay in rfPIBF, raPIBF, or rcPIBF-treated HUAEC (**i**) and quantified results of their migration (**j**) and tube forming assay (**k**). **l** Migration assay in rfPIBF, raPIBF, or rcPIBF-treated Pl-PC. Migration (**g**, **j**, and **l**) and tube formation data (**h**, **k**) are expressed relative to that of control and presented as mean ± SEM; *P < 0.05; **P < 0.01; ***P < 0.001; ****P < 0.0001 in one-way ANOVA. Source data are provided as a Source Data file.

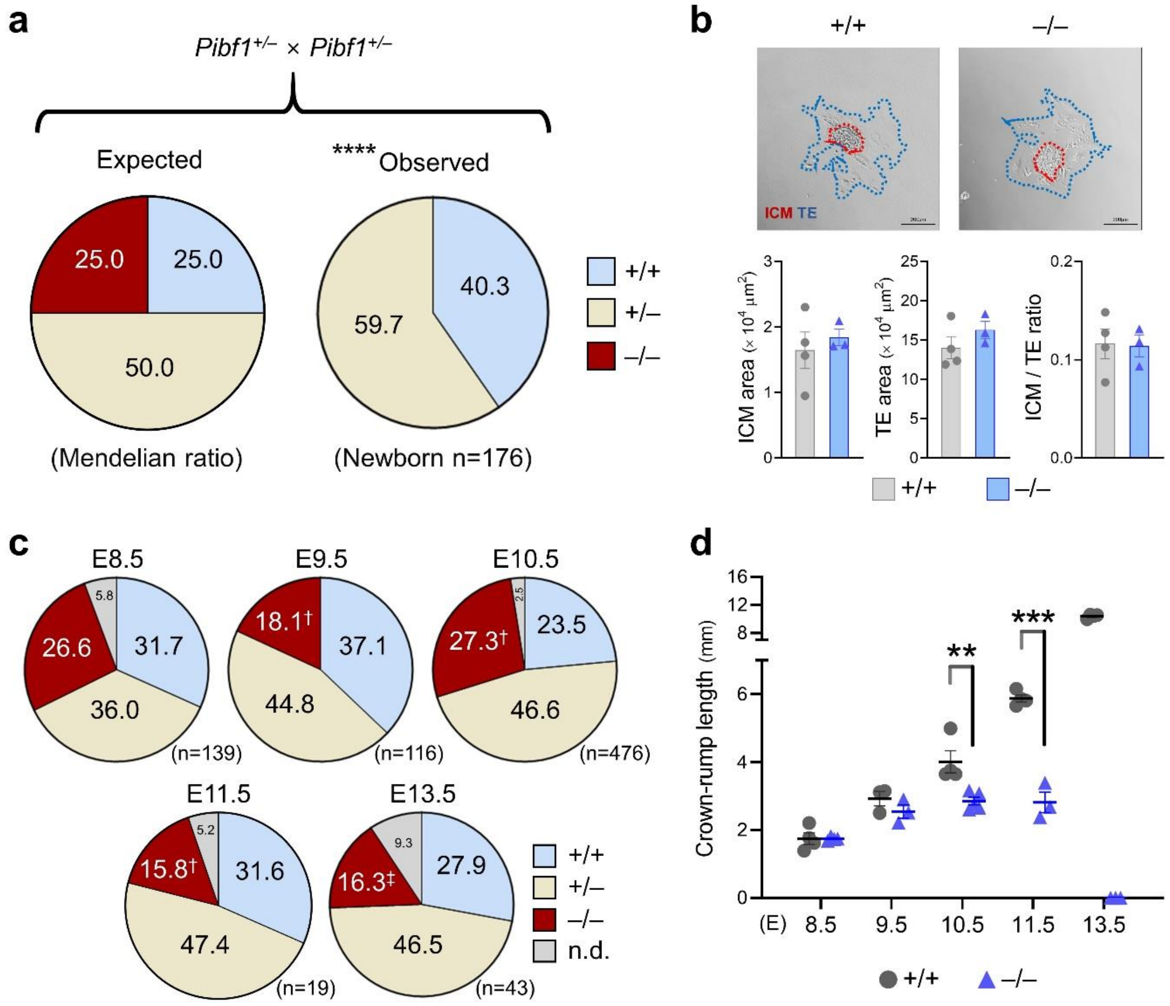

Supplementary Fig. 7. *Pibf1*-null mice display *in utero* lethality, and its time window of impaired embryogenesis is associated with the primary placentation period.
a The expected and observed genotype distribution in the newborn after *Pibf1*$^{+/-}$ intercross. ****$P < 0.0001$ in χ^2 test. **b** Quantification of the area of inner cell mass (ICM), trophectoderm (TE), and their ratio (ICM/TE) as determined by outgrowth culture of *Pibf1*$^{-/-}$ embryos. Data are presented as mean ± SEM. **c** Genotype distribution of *Pibf1*$^{+/-}$ intercross at E8.5-13.5. †Developmentally delayed with several defects (reduced size, pericardial effusion, failed tail turning). ‡Almost resorbed with few remnants. **d** Measurement of the crown-rump length of *Pibf1*$^{-/-}$ embryos at E8.5-13.5. Data are presented as mean ± SEM; **$P < 0.05$; ***$P < 0.01$ in Student's t-test. Note that the crown-rump length of *Pibf1*$^{-/-}$ embryos was considered zero at E13.5 and was not compared statistically to the WT group because *Pibf1*$^{-/-}$ embryos at this stage were almost resorbed with few remnants, which were used for genotyping. Source data are provided as a Source Data file.

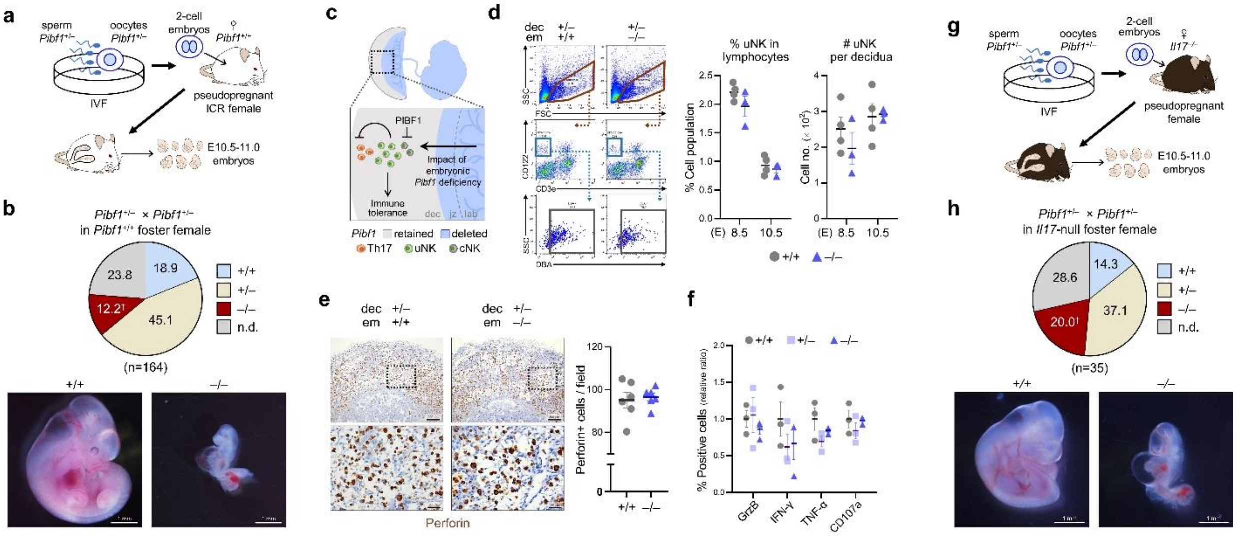

Supplementary Fig. 8. Viability of *Pibf1*-null embryos is not associated with the maternal genetic dose of *Pibf1* or uterine immune tolerance regarding natural killer (uNK) cells/lymphocytes.

a *In vitro* fertilization (IVF) and embryo transfer (ET) of *Pibf1*$^{+/-}$ intercrossed embryos to wild-type pseudopregnant female mice. **b** Genotype distribution of E10.5 embryos in wild-type mothers after *Pibf1*$^{+/-}$ intercross (upper) and whole mount images of their *Pibf1*$^{-/-}$ embryo (lower). †Developmentally delayed with several defects (reduced size, pericardial effusion, failed tail turning). **c** Schematic diagram of the lineages containing *Pibf1* deletion in *Pibf1*$^{-/-}$ conceptus and immune tolerance regulated by a balance between uterine NK (uNK) and helper T 17 (Th17) cells in the decidua. **d** Fluorescence-activated cell sorting (FACS) analysis of CD3e^{-}CD122^{+}DBA^{+} uNK cells in the *Pibf1*$^{-/-}$ conceptus mesometrial region at E8.5 and E10.5. Data are presented as mean ± SEM. **e** Quantification of Perforin-positive uNK cells in the *Pibf1*$^{-/-}$ conceptus decidual region. Data are presented as mean ± SEM. **f** FACS analysis of granzyme B, interferon-γ, TNF-α, or CD107a positive uNK cells at E10.5. Data are expressed relative to that of WT and presented as mean ± SEM. **g** IVF and ET of *Pibf1*$^{+/-}$ intercrossed embryos to *Il17*-deficient pseudopregnant female mice. **h** Genotype distribution of E10.5 embryos in *Il17*-deficient mothers after *Pibf1*$^{+/-}$ intercross (upper) and whole mount images of their *Pibf1*$^{-/-}$ embryo (lower). †Developmentally delayed with several defects (reduced size, pericardial effusion, failed tail turning). Source data are provided as a Source Data file.

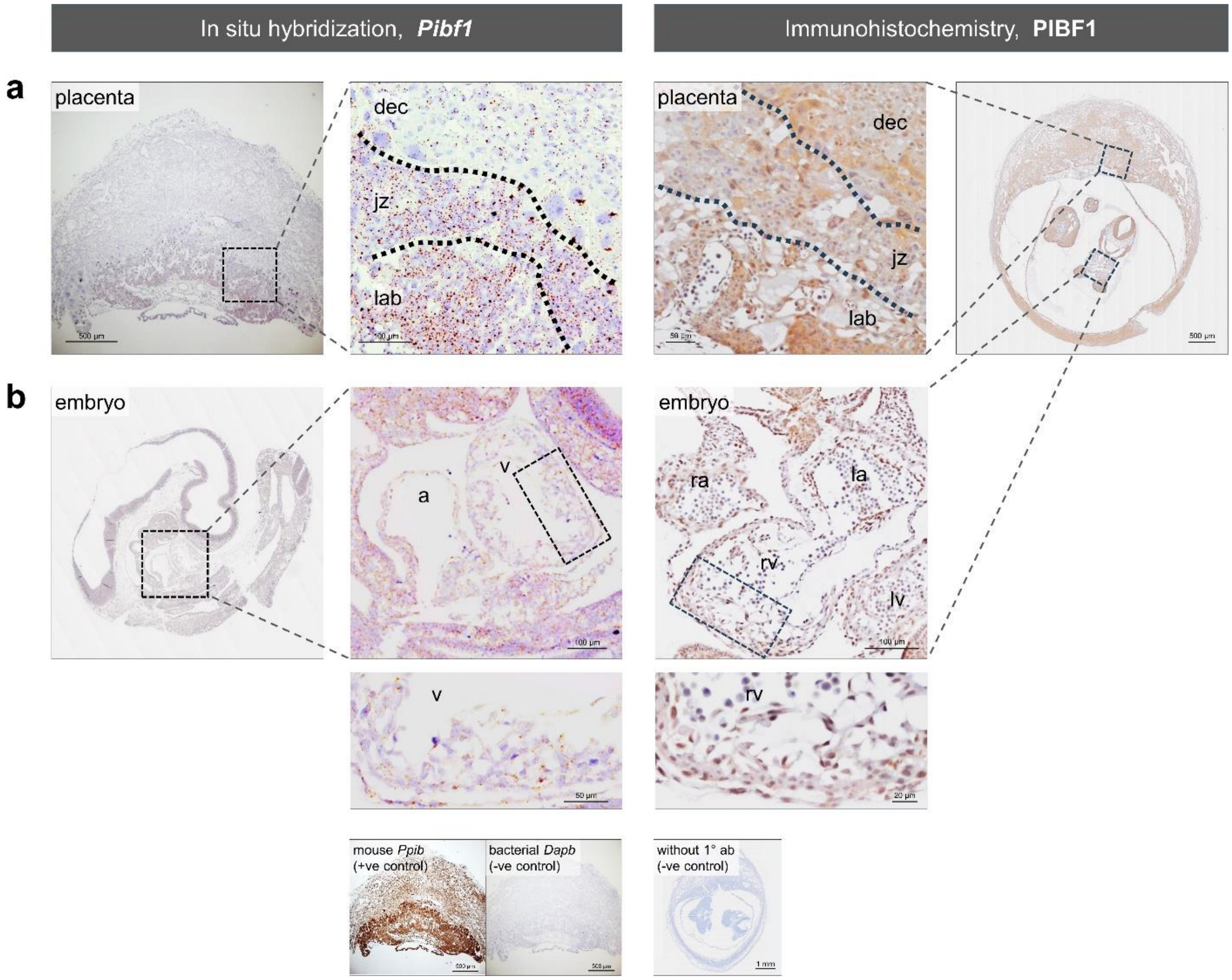

Supplementary Fig. 9. *In situ* hybridization (ISH) and immunohistochemistry (IHC) assays of Pibf1 in the mouse placenta and embryo at E10.5.

a, b Note that the brown dots in ISH or brown-colored positive signals in IHC were ubiquitously present throughout the placenta (**a**) and embryo (**b**). Probes detecting mouse *Ppib* and bacterial *dapb* were used as positive and negative controls in the ISH assay, respectively. Sections without a primary antibody were used as a negative control in the IHC assay. a, atrium. v, ventricle. dec, decidua. jz, junctional zone. lab, labyrinth. ra and la, right and left atrium. rv and lv, right and left ventricle.

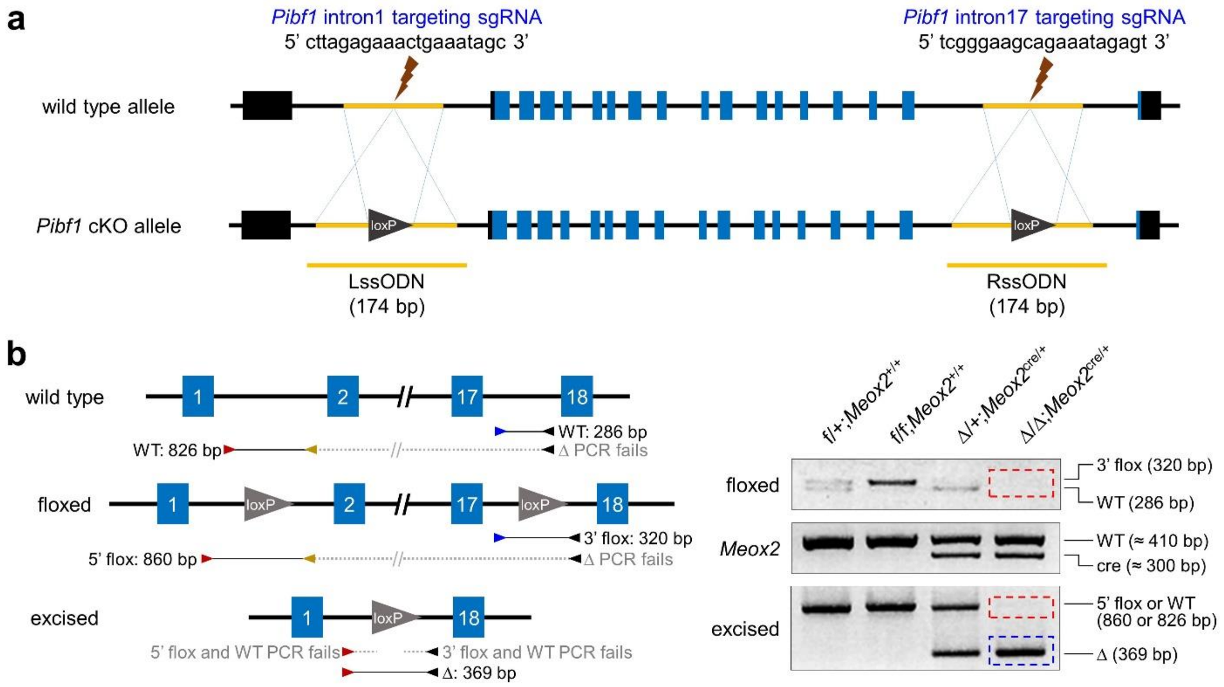

Supplementary Fig. 10. Epiblast-specific *Pibf1* knockout using a *Meox2*-driven cre line.

a Generation of *Pibf1* conditional knockout (cKO) mice using CRISPR/Cas9. **b** Genotyping to verify *Meox2*^{cre}-mediated *Pibf1* deletion. Genomic DNA samples prepared from the tail of E13.5 fetus were used as a template for PCR followed by agarose gel electrophoresis assay. Primer locations are indicated with black or colored arrowheads on relevant alleles. Colored dotted boxes indicate the absence of wild-type or *Pibf1* floxed allele (red) and the presence of its recombined allele (blue) in the *Pibf1* cKO mutant. Source data are provided as a Source Data file.

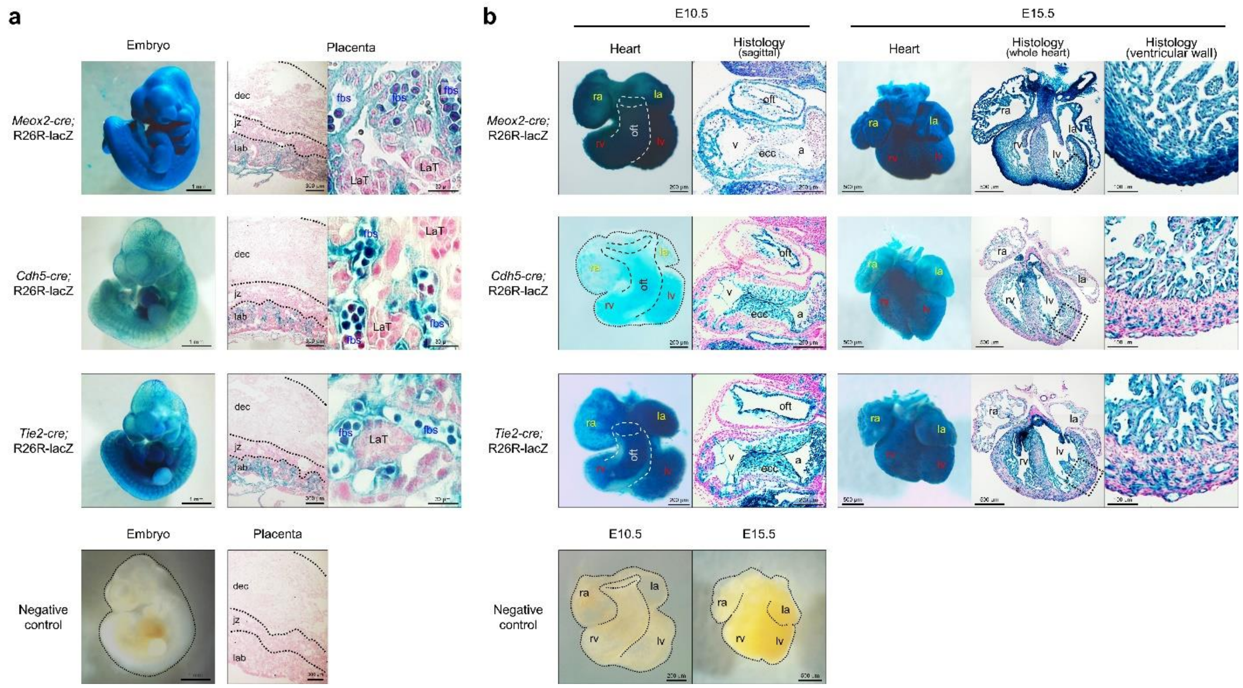

Supplementary Fig. 11. *Meox2-*, *Cdh5-* or *Tie2*-driven cre recombinase activity in the placenta (a) and the embryonic heart (b).

Males carrying *Meox2*-, *Cdh5*-, or *Tie2*-cre allele were mated with female R26R-lacZ reporters, and embryos, placentas, or hearts were obtained at E10.5 or E15.5, subjected to whole-mount X-gal staining. Embryos carrying R26R-lacZ but negative for *cre* allele were used as a negative control. dec, decidua. jz, junctional zone. lab, labyrinth. fbs, fetal blood space. LaT, labyrinth trophoblasts. a, atrium. v, ventricle. ra and la, right and left atrium. rv and lv, right and left ventricle. oft, outflow tract. ecc, endocardial cushion.

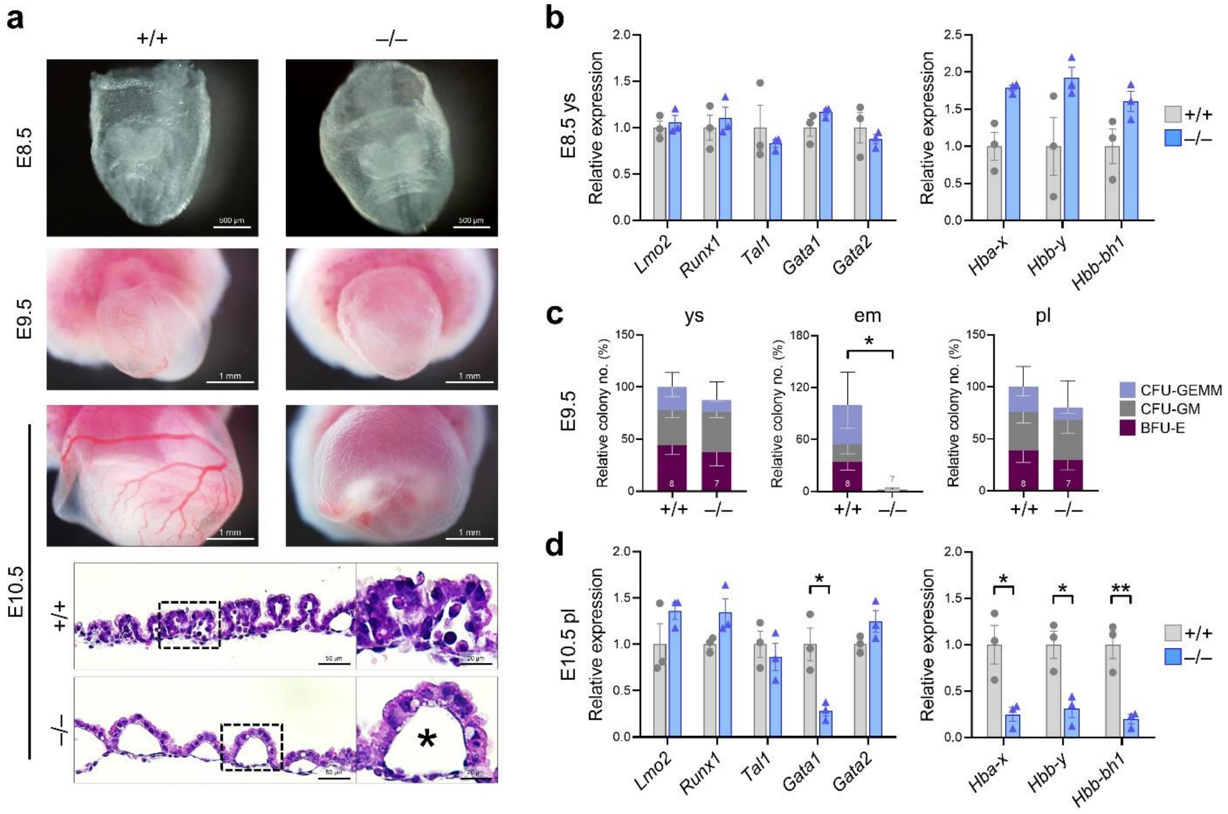

Supplementary Fig. 12. Supporting data for defective developing hematopoietic tissues of *Pibf1*-null conceptus.

a Whole-mount images of *Pibf1*$^{-/-}$ yolk sac (YS) at E8.5-10.5 and histological findings of *Pibf1*$^{-/-}$ YS at E10.5. Note the reduced vasculature and the pale appearance in the whole-mount image of *Pibf1*$^{-/-}$ YS at E10.5. Asterisk indicates blood islands that were dilated and disorganized and contained few erythroid cells in *Pibf1*$^{-/-}$ E10.5 YS. **b** Expressions of markers for hematopoiesis (left) and globin in primitive erythroid cells (right) in *Pibf1*$^{-/-}$ YS at E8.5. **c** Hematopoietic potential in *Pibf1*$^{-/-}$ yolk sac (ys), embryo proper (em), and placenta (pl) at E9.5 as determined by colony forming unit (CFU) assay. Data are expressed relative to that of wild type (WT). The number in each bar indicates sample size (n). CFU-GEMM = CFU-granulocyte, erythroid, macrophage, megakaryocyte. CFU-GM = CFU-granulocyte, macrophage. BFU-E = burst forming unit-erythroid. **d** Expressions of markers for hematopoiesis (left) and globin in primitive erythroid cells (right) in *Pibf1*$^{-/-}$ placenta at E10.5. Transcript expression data (**b** and **d**) were normalized using *Gapdh* and expressed relative to that of WT. Data are presented as mean ± SEM (**b-d**); *$P < 0.05$; **$P < 0.01$ in Student's *t*-test. Source data are provided as a Source Data file.

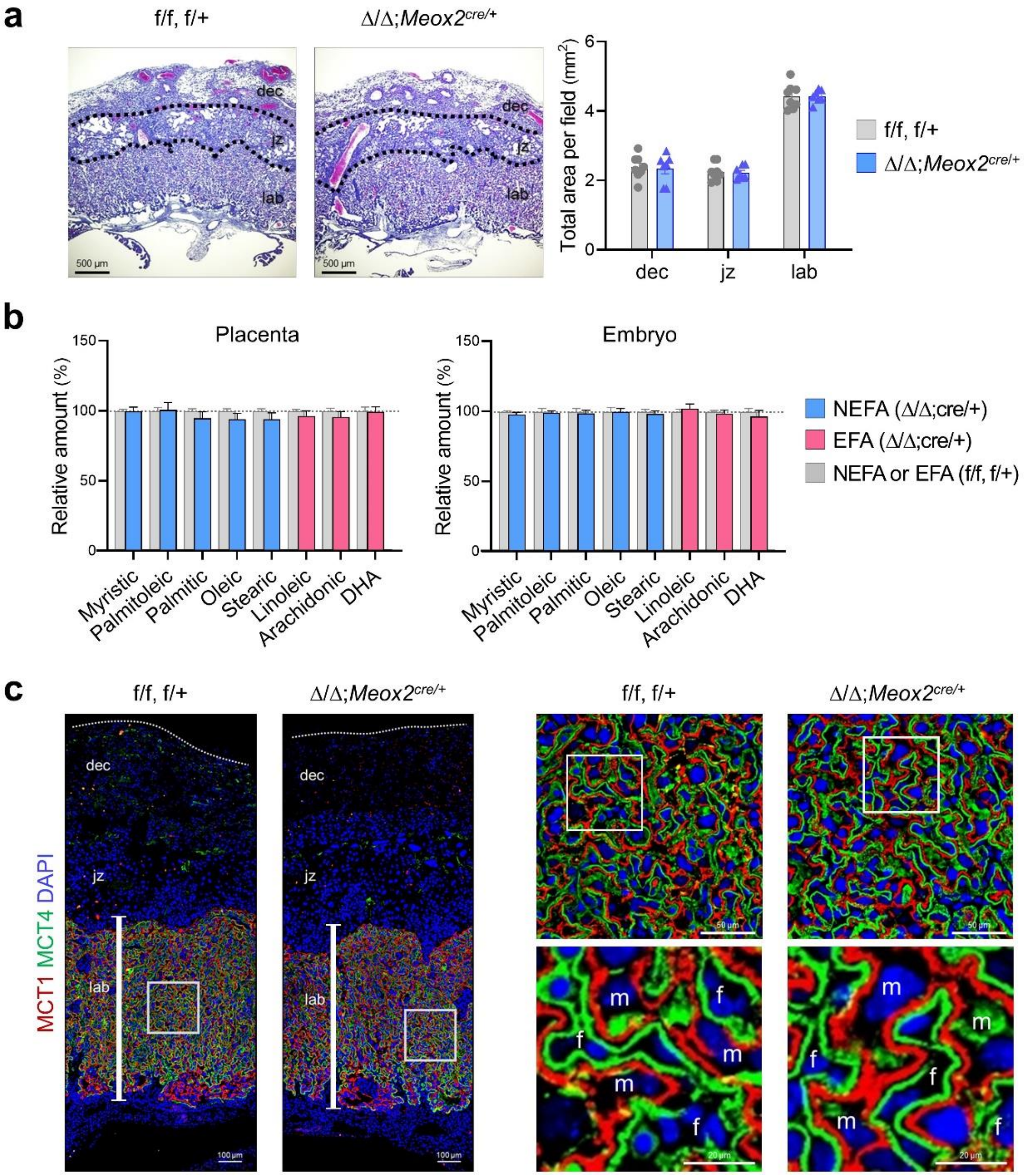

Supplementary Fig. 13. Supporting data for *Pibf1*$^{\Delta/\Delta}$;*Meox2*$^{cre/+}$ placenta phenotype.
a Gross histological assessment of *Meox2*^{cre}-mediated *Pibf1* cKO (cKO^{Meox2}) placentas at E13.5. Data are presented as mean ± SEM. **b** Fatty acid (FA) contents in cKO^{Meox2} placenta and embryo at E13.5 as measured by gas chromatography coupled with mass spectrometry (GC-MS). Data were expressed as a relative amount (%) (colored bar) as compared to that of WT (gray bar) (n=6/genotype). The term "acid" was omitted except for docosahexaenoic acid (DHA). NEFA, non-essential FA; EFA, essential FA. **c** Immunofluorescence staining of MCT1 (red) and MCT4 (green) to detect SynT-I and -II layers, respectively, in cKO^{Meox2} placenta at E13.5. m and f, maternal and fetal blood sinus, respectively. Source data are provided as a Source Data file.

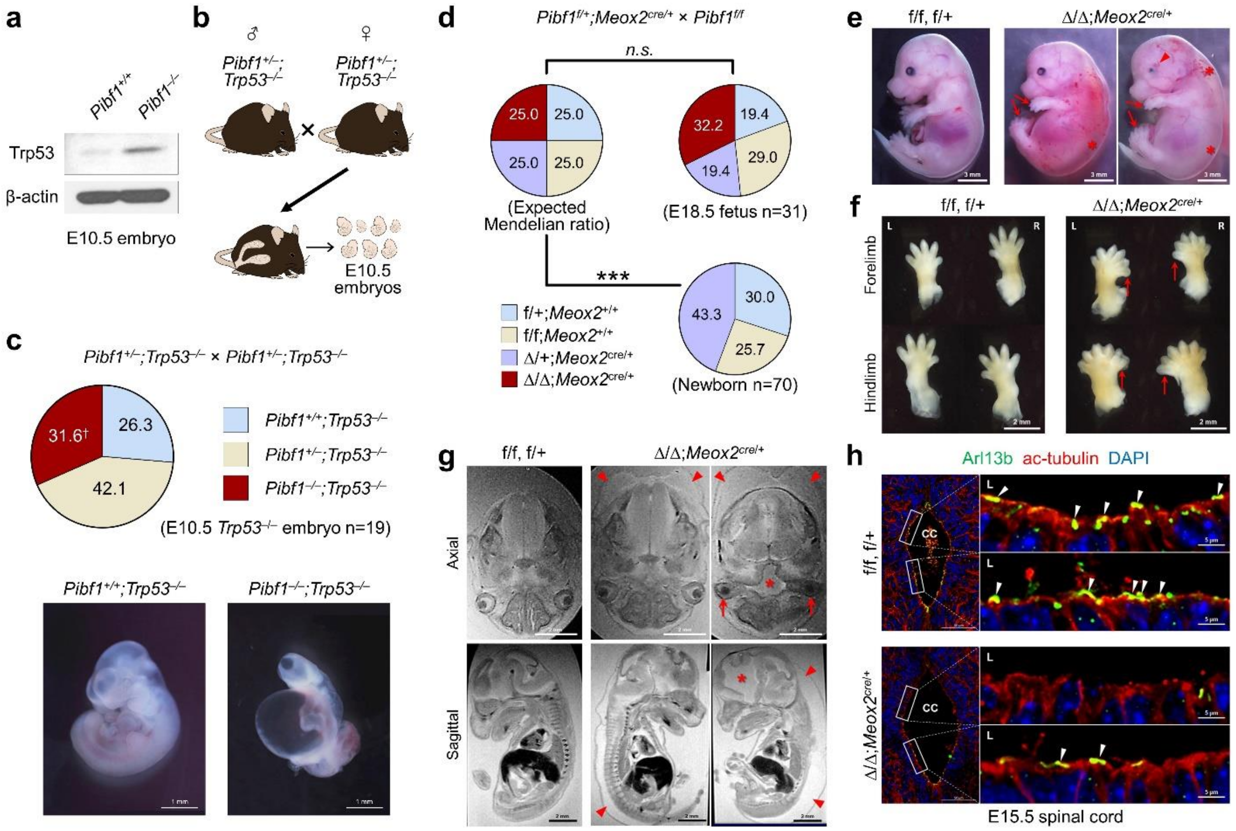

Supplementary Fig. 14. *Pibf1*-null embryonic lethality is not *Trp53*-dependent, and ciliopathy-related anomalies are seen in *Pibf1*$^{\Delta/\Delta}$;*Meox2*$^{cre/+}$ fetuses at late gestation.

a Expression of Trp53 in E10.5 *Pibf1*$^{-/-}$ embryo. β-actin was used as an internal control. **b** Mating scheme to obtain *Pibf1*$^{-/-}$;*Trp53*$^{-/-}$ embryos. **c** Genotype distribution of *Pibf1*$^{+/-}$ intercrossed E10.5 embryos deficient with *Trp53* (upper panel) and whole mount images of their *Pibf1*$^{-/-}$;*Trp53*$^{-/-}$ embryo (lower panel). †Developmentally delayed with several defects (reduced size, pericardial effusion, failed tail turning). **d** The expected and observed genotype distribution of cKO^{Meox2} intercross at E18.5 and after birth; ***$P < 0.001$ in χ^2 test. **e, f** Gross morphology of cKO^{Meox2} E15.5 fetuses (**e**) and magnified views of their limbs (**f**). Arrow, arrowhead, and asterisk indicate polydactyly, microphthalmia, and subcutaneous edema. **g** Magnetic resonance imaging of cKO^{Meox2} E15.5 fetuses. Arrow, arrowhead, and asterisk indicate microphthalmia, subcutaneous edema, and hydrocephalus. **h** Immunofluorescence staining for detecting Arl13b/acetylated tubulin dual-positive primary cilia (arrowheads) in the spinal cord of cKO^{Meox2} E15.5 fetus. cc, central canal. L, lumen. Source data are provided as a Source Data file.

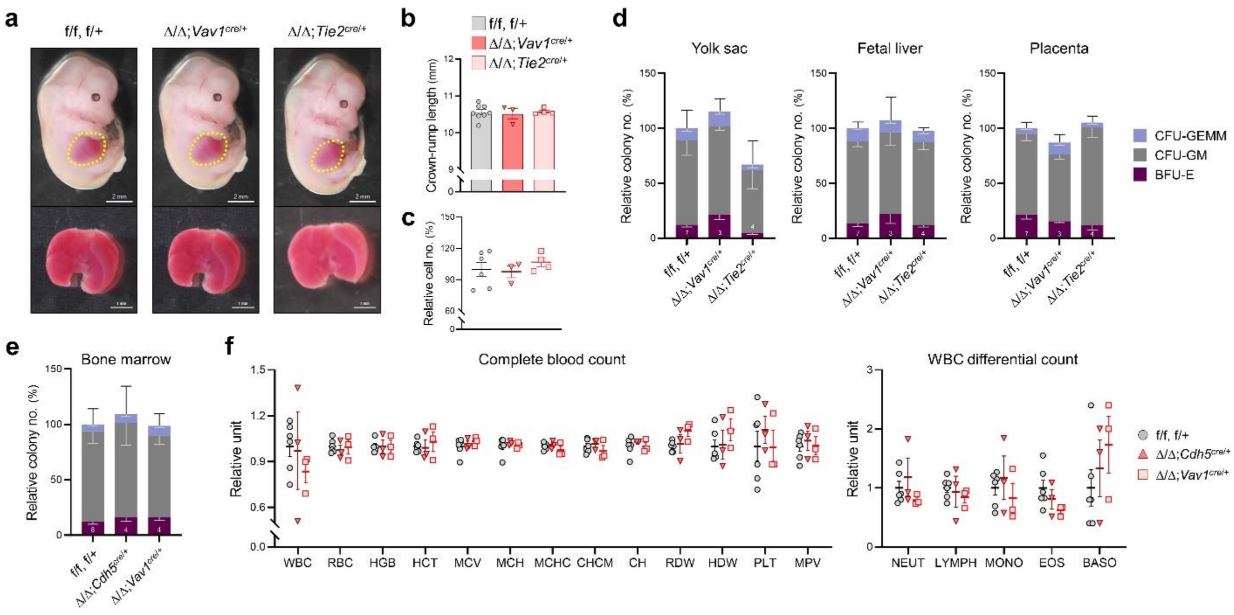

Supplementary Fig. 15. Loss of *Pibf1* in hematopoietic lineages does not disrupt normal hematopoiesis.

a Gross images of fetus and fetal liver of hematopoiesis-specific *Pibf1* cKO using *Vav1*-cre (cKO^{Vav1}) or *Tie2*-cre (cKO^{Tie2}) line at E13.5. Yellow dotted lines indicate the outline of the fetal liver. **b** Measurement of the crown-rump length of cKO^{Vav1} and cKO^{Tie2} E13.5 fetuses. **c** Analysis of the cellularity from E13.5 fetal livers of cKO^{Vav1} and cKO^{Tie2}. **d** CFU assay of the yolk sac, fetal liver, and placenta of cKO^{Vav1} and cKO^{Tie2} at E13.5. **e, f** CFU assay of bone marrow (**e**) and complete blood count with white blood cell differential (**f**) in cKO^{Cdh5} and cKO^{Vav1} adult mice (4-month-old). Data are expressed relative to the control group and presented as mean ± SEM in **c**-**f**. The number in each bar indicates sample size (n) in **d** and **e**. Source data are provided as a Source Data file.

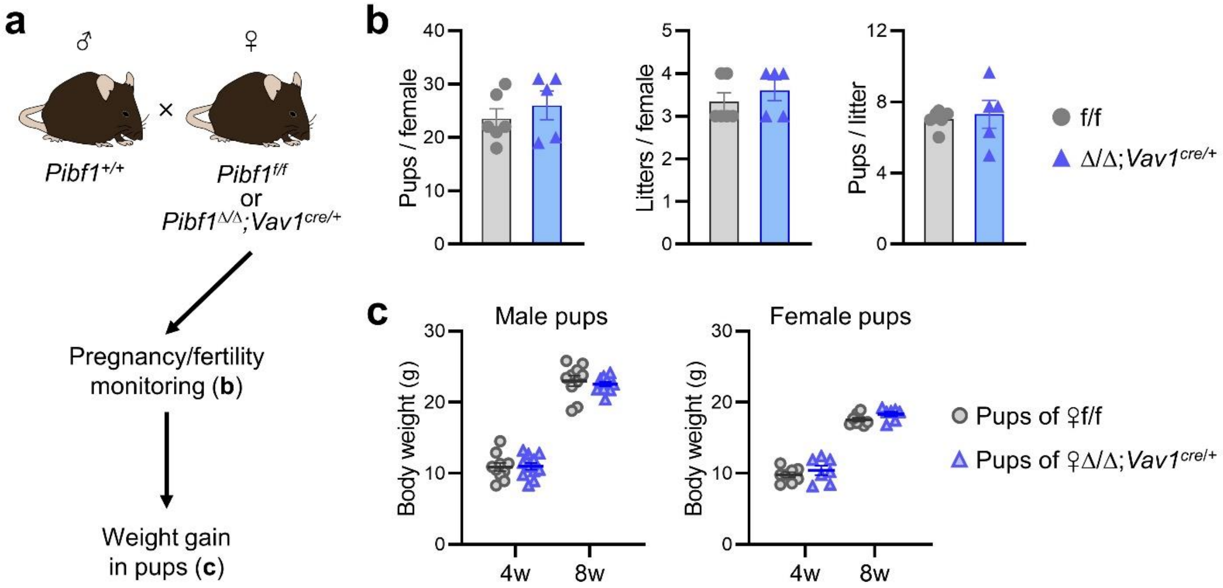

Supplementary Fig. 16. Deficiency of *Pibf1* in maternal blood and immune cells does not disrupt normal fertility.

a Mating scheme to monitor pregnancy and outcome. *Pibf1*$^{f/f}$ or cKO^{Vav1} females (8-week-old) were mated with wild-type males for 4 months (**b**), and the resulting pups from three different litters per genotype were monitored with body weight for eight weeks (**c**). Source data are provided as a Source Data file.

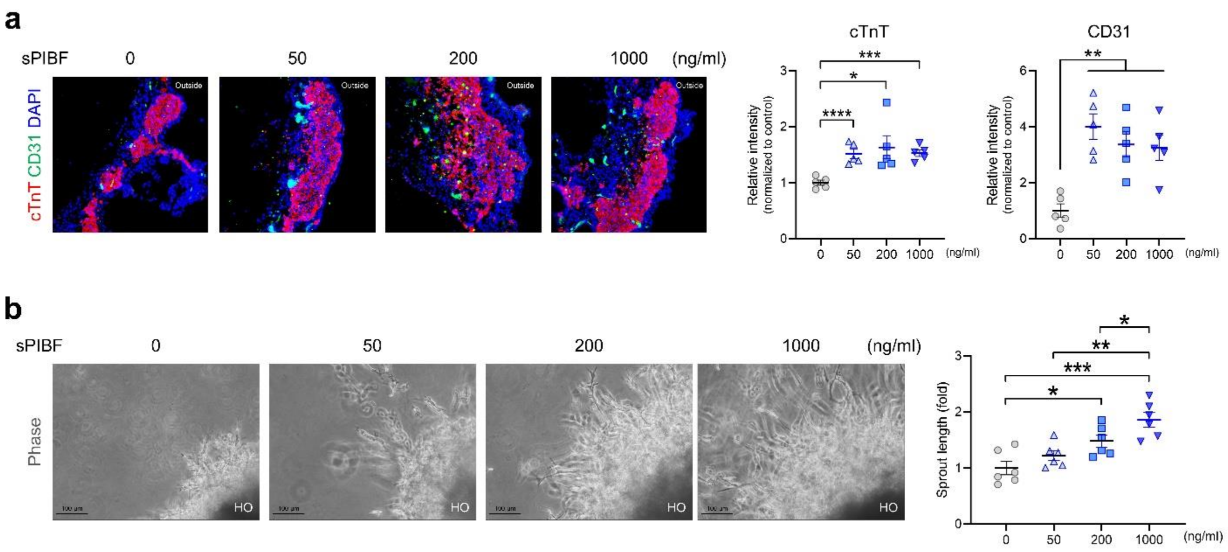

Supplementary Fig. 17. Dose effect of sPIBF1 on hHO development.

a, **b** Immunostaining of cTnT and CD31 (**a**) and sprouting assay (**b**) in hHOs treated with rsPIBF at doses of 50, 200, and 1000 ng/ml. The intensity of fluorescence-labeled cells (**a**) and sprout length data (**b**) are expressed relative to control. Data are presented as mean ± SEM. **P*<0.05; ***P*<0.01; ****P*<0.001; *****P*<0.0001 in one-way ANOVA. Source data are provided as a Source Data file.

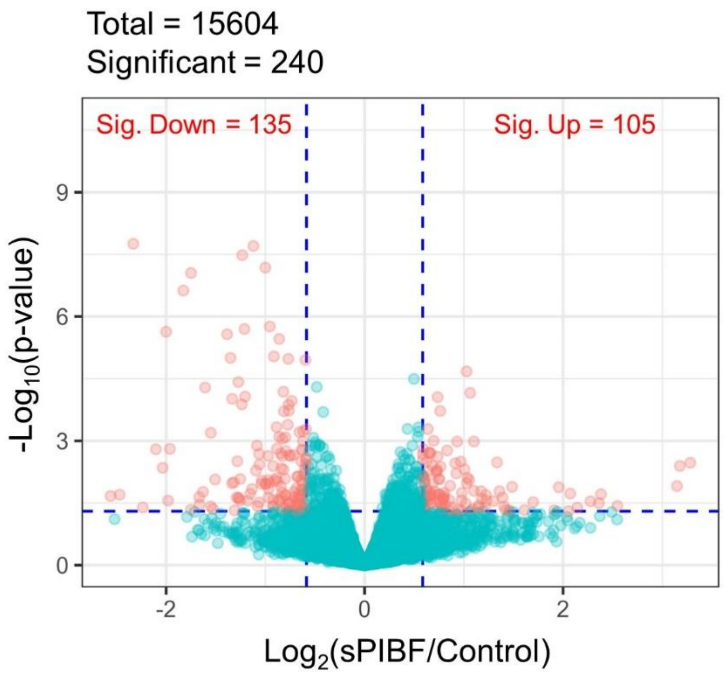

Supplementary Fig. 18. Volcano plot of all genes expressed in rsPIBF-treated human heart organoids.

All expressed genes are plotted on a log2 scale on the X-axis and the calculated probability (p-value) on a -log10 scale on the Y-axis. Red dots indicate the differentially expressed genes (DEGs) significantly up- or down-regulated with a fold change >1.5 at an adjusted p-value <0.05. Vertical and horizontal dotted blue lines indicate the threshold.
