# Supplementary material for: PIBF1 regulates trophoblast syncytialization and promotes cardiovascular development
Source: Nat Commun. 2024 Feb 19;15:1487. doi: 10.1038/s41467-024-45647-8 (PMC10876648; doi:10.1038/s41467-024-45647-8)
Supplement: Supplementary file 3 — Description of Additional Supplementary Files [file 41467_2024_45647_MOESM3_ESM.pdf]

Description of Additional Supplementary Files

File Name: Supplementary Data 1
Description: Fatty acid contents in *Pibf1* KO placenta and embryo at E9.5-10.5 as measured by GC-MS.

File Name: Supplementary Data 2
Description: Fatty acid contents in *Meox2cre*-mediated Pibf1 cKO placenta and embryo at E13.5 as measured by GC-MS.

File Name: Supplementary Data 3
Description: GO enrichment analysis of up-regulated DEGs in sPIBF-treated human heart organoids.

File Name: Supplementary Data 4
Description: GO enrichment analysis of down-regulated DEGs in sPIBF-treated human heart organoids.

File Name: Supplementary Data 5
Description: List of primers used for genotyping and quantitative RT-PCR.

File Name: Supplementary Data 6
Description: List of antibodies used for immunofluorescence staining, FACS, and Western blotting.
