# Supplementary material for: PIBF1 regulates trophoblast syncytialization and promotes cardiovascular development
Source: Nat Commun. 2024 Feb 19;15:1487. doi: 10.1038/s41467-024-45647-8 (PMC10876648; doi:10.1038/s41467-024-45647-8)
Supplement: Supplementary file 10 — Reporting Summary [file 41467_2024_45647_MOESM10_ESM.pdf]

nature portfolio

Corresponding author(s): In-Jeoung Baek and You Sook Cho

Last updated by author(s): Jan 27, 2024

Reporting Summary

Nature Portfolio wishes to improve the reproducibility of the work that we publish. This form provides structure for consistency and transparency in reporting. For further information on Nature Portfolio policies, see our Editorial Policies and the Editorial Policy Checklist.

Statistics

For all statistical analyses, confirm that the following items are present in the figure legend, table legend, main text, or Methods section.

n/a	Confirmed	
☐	☒	The exact sample size (*n*) for each experimental group/condition, given as a discrete number and unit of measurement
☐	☒	A statement on whether measurements were taken from distinct samples or whether the same sample was measured repeatedly
☐	☒	The statistical test(s) used AND whether they are one- or two-sided<br>*Only common tests should be described solely by name; describe more complex techniques in the Methods section.*
☒	☐	A description of all covariates tested
☐	☒	A description of any assumptions or corrections, such as tests of normality and adjustment for multiple comparisons
☐	☒	A full description of the statistical parameters including central tendency (e.g. means) or other basic estimates (e.g. regression coefficient) AND variation (e.g. standard deviation) or associated estimates of uncertainty (e.g. confidence intervals)
☐	☒	For null hypothesis testing, the test statistic (e.g. F, t, r) with confidence intervals, effect sizes, degrees of freedom and P value noted<br>*Give P values as exact values whenever suitable.*
☒	☐	For Bayesian analysis, information on the choice of priors and Markov chain Monte Carlo settings
☒	☐	For hierarchical and complex designs, identification of the appropriate level for tests and full reporting of outcomes
☒	☐	Estimates of effect sizes (e.g. Cohen's *d*, Pearson's *r*), indicating how they were calculated

Our web collection on statistics for biologists contains articles on many of the points above.

Software and code

Policy information about availability of computer code

Data collection	Confocal imaging experiments: Zeiss LSM 880 (Carl Zeiss).<br>Multi-electrode array: MEA device (Maestro Edge version, Axion BioSystems)<br>Whole-mount sample images: ProgRes C5 camera (Jenoptik Optical Systems)<br>Multiple reaction monitoring: 1290 Infinity LC and 6495 triple quadrupole LC/MS (Agilent Technologies)<br>FACS: FACSCanto II system (BD Biosciences)<br>Transmission electron microscope: H-7100 TEM (Hitachi)<br>qRT-PCR: QuantStudio3 Real-time PCR system (Applied Biosystems)<br>MRI imaging: Preclinical MRI scanner (Bruker Inc.)
Data analysis	Image data analysis: ImageJ software version 1.54d (NIH), Capture Pro software version 1.10.0.1 (Jenoptik Optical Systems)<br>Multi-electrode array: Cardiac analysis Tool and AxIS metric Plotting Tool (Axion BioSystems)<br>Multiple reaction monitoring: Skyline software (version 19.1.0.193, University of Washington, MacCoss Lab)<br>FACS: FlowJo software (version 10.2, Tree Star)<br>MRI imaging in fetuses: Paravision 6.0 (Bruker Inc.), AsanJ-Morphometry (a dedicated software for ImageJ (NIH))<br>Statistics: GraphPad Prism 9 (version 9.5.1, GraphPad software)

For manuscripts utilizing custom algorithms or software that are central to the research but not yet described in published literature, software must be made available to editors and reviewers. We strongly encourage code deposition in a community repository (e.g. GitHub). See the Nature Portfolio guidelines for submitting code & software for further information.

Data

Policy information about availability of data

All manuscripts must include a data availability statement. This statement should provide the following information, where applicable:

- Accession codes, unique identifiers, or web links for publicly available datasets
- A description of any restrictions on data availability
- For clinical datasets or third party data, please ensure that the statement adheres to our policy

The RNA-seq data for rsPIBF-treated hHO generated in this study have been deposited in the GEO database under accession code GSE229265 at https://www.ncbi.nlm.nih.gov/geo/query/acc.cgi?acc=GSE229265. All other data are available within the article and its Supplementary Information files. Source data are provided with this paper.

Research involving human participants, their data, or biological material

Policy information about studies with human participants or human data. See also policy information about sex, gender (identity/presentation), and sexual orientation and race, ethnicity and racism.

Reporting on sex and gender	This study included pregnant women.
Reporting on race, ethnicity, or other socially relevant groupings	Donor demographic (race, ethnicity) data was not available and was not included in this study.
Population characteristics	n/a
Recruitment	Umbilical cord donors were recruited from pregnant women who participated in a pregnancy at Asan Medical Center in Seoul, Republic of Korea. There was no bias in selecting donors for recruitment in clinically normal pregnant conditions.
Ethics oversight	The collection and use of human materials for research purposes were approved by the Institutional Review Board of the Asan Medical Center (approval ID: 2022-0664).

Note that full information on the approval of the study protocol must also be provided in the manuscript.

Field-specific reporting

Please select the one below that is the best fit for your research. If you are not sure, read the appropriate sections before making your selection.

☒ Life sciences ☐ Behavioural & social sciences ☐ Ecological, evolutionary & environmental sciences

For a reference copy of the document with all sections, see nature.com/documents/nr-reporting-summary-flat.pdf

Life sciences study design

All studies must disclose on these points even when the disclosure is negative.

Sample size	We used sample sizes in previously reported literature, including in vivo or in vitro studies in this field (Nat Commun 2017, 8(1):352; eLife 2021, 25:10:e63254; Nat Commun 2023, 14(1):1174) or our preliminary studies. In vitro samples were collected until statistical significance was reached. The sample size of animal experiments (dams from mother) was chosen according to similar studies in the field, and conceptus samples with interested genotypes were obtained from at least four pregnant mice.
Data exclusions	No data was excluded.
Replication	Experiments were replicated at least two times, as mentioned in the methods section, and all experiments in the article were reliably reproduced.
Randomization	Cells grown under the same conditions were randomly allocated into different groups without bias. Mice (in pregnancy or not) were randomly assigned to groups with different experiments and euthanized at the designated time.
Blinding	Experimental animals were randomized throughout the study, and information on genotype and study design was blinded to the technicians or the core facilities. All other investigators were unblinded because the procedures of the experiments performed in this study were not susceptible to bias.

Reporting for specific materials, systems and methods

We require information from authors about some types of materials, experimental systems and methods used in many studies. Here, indicate whether each material, system or method listed is relevant to your study. If you are not sure if a list item applies to your research, read the appropriate section before selecting a response.

Materials & experimental systems

n/a	Involved in the study
☐	☒ Antibodies
☐	☒ Eukaryotic cell lines
☒	☐ Palaeontology and archaeology
☐	☒ Animals and other organisms
☒	☐ Clinical data
☒	☐ Dual use research of concern
☒	☐ Plants

Methods

n/a	Involved in the study
☒	☐ ChIP-seq
☐	☒ Flow cytometry
☒	☐ MRI-based neuroimaging

Antibodies

Antibodies used

Antibodies for immunofluorescence staining
primary antibodies: β-hCG (Abcam, ab53087, 1/100), HLA-G (Abcam, ab7759, 1/100), KRT7 (Abcam, ab181598, 1/1000), GATA3 (Cell Signaling Technology, 5852, 1/200), TEAD4 (Abcam, ab58310, 1/100), TP63 (Cell Signaling Technology, 13109, 1/100), cTnT (Abcam, ab45932, 1/200), CD31 (Millipore, CBL468, 1/200), PDGFRβ (Abcam, ab32570, 1/200), MCT1 (Sigma, AB1286-I, 1/150), MCT4 (Santa Cruz, sc-376140, 1/150), CD31 (Abcam, ab28364, 1/200), cTnT (Abcam, ab209813, 1/500), ARL13B (Proteintech, 17711-1-AP, 1/500), Acetylated Tubulin (Proteintech, 66200-1-Ig, 1/1000)
secondary antibodies: Anti-chicken IgY, AF488 (Invitrogen, A11039, 1/200), Anti-mouse IgG, AF594 (Invitrogen, A11005, 1/200), Anti-mouse IgG, AF488 (Invitrogen, A11001, 1/200), Anti-rabbit IgG, AF488 (Invitrogen, A11008, 1/200), Anti-rabbit IgG, AF555 (Invitrogen, A21429, 1/200)

FACS
FACS for uNK population: FITC-conjugated Dolichos Biflorus Agglutinin (Invitrogen, L32474, 1/100), anti-CD3e-PerCP-Cy5.5 (BD Biosciences, 551163, 1/100), anti-CD122-PE (eBioscience, 12-1221-82, 1/100)
FACS for uNK function: anti-CD3-Brilliant Violet 605 (BioLegend, 100237, 1/200), anti-CD122-APC-Cy7 (BioLegend, 123221, 1/200), anti-Granzyme B-Pacific Blue (BioLegend, 515407, 1/200), anti-TNF-α-FITC (BioLegend, 506303, 1/200), anti-IFN-γ-APC (BioLegend, 505809, 1/200), anti-CD107a-PE (BioLegend, 121611, 1/200)

Western blotting
primary antibodies: β-hCG (Abcam, ab54410, 1/1000), PIBF1 (provided by Szekeres-Bartho, J., PMID:14634107, 1/1000), Phospho-PI3K p85 (Cell Signaling Technology, 4228, 1/1000), PI3K p85 (Cell Signaling Technology, 4257, 1/1000), Phospho-AKT (Cell Signaling Technology, 4060, 1/1000), AKT (Cell Signaling Technology, 4691, 1/1000), Phospho-eNOS (Cell Signaling Technology, 9571, 1/1000), eNOS (Cell Signaling Technology, 32027, 1/1000), Phospho-p38 MAPK (Cell Signaling Technology, 4511, 1/1000), p38 MAPK (Santa Cruz, sc7972, 1/1000), Phospho-p44/42 MAPK (ERK1/2) (Cell Signaling Technology, 4377, 1/1000), p44/42 MAPK (ERK1/2) (Cell Signaling Technology, 4695, 1/1000), Phospho-STAT3 (Cell Signaling Technology, 9145, 1/1000)
STAT3 (Cell Signaling Technology, 9139, 1/1000), TRP53 (Santa Cruz, sc6243, 1/1000), β-ACTIN (Santa Cruz, sc47778, 1/1000)
secondary antibodies: anti-mouse IgG, HRP-linked (Cell Signaling Technology, 7076s, 1/5000), anti-rabbit IgG, HRP-linked (Cell Signaling Technology, 7074s, 1/5000)

All the primary and secondary antibodies are listed in the reporting summary as referenced in the manuscript and supplementary table 4. Also, the information on all the antibodies listed here is provided in supplementary table 4.

Validation

The antibodies were validated with their correct species, target specificity, and applicable usages as described in the datasheet of respective manufacturers or by us, as shown in the manuscript. The antibody against PIBF1 was generously provided by Szekeres-Bartho, J. (source, PMID:14634107). The other antibodies were all sourced commercially with independent validations.

Eukaryotic cell lines

Policy information about cell lines and Sex and Gender in Research

Cell line source(s)

BeWo cells (CCL-98, ATCC), HUVEC (isolated from the human umbilical cord of pregnant women)
HUAEC (8010, ScienCell Research Laboratories), Human pericytes from the placenta (Pl-PC; C-12980, PromoCell), Human trophoblast stem (hTS) cells (RCB4936, Riken BRC)

All the cell lines listed here in the reporting summary are also listed in the methods section of the manuscript.

Authentication

The KO lines (BeWo, hTS) were authenticated with protein expression and Sanger sequencing. All cell lines without stimulation or treatment grew as expected with appropriate morphology.

Mycoplasma contamination

All cell lines were tested to be negative for mycoplasma contamination.

Commonly misidentified lines
(See ICLAC register)

No commonly misidentified cell lines were used in this study.

Animals and other research organisms

Policy information about studies involving animals; ARRIVE guidelines recommended for reporting animal research, and Sex and Gender in Research

Laboratory animals	Pibf1 (Pibf1em1Hwl/J, PMID:23302927), Trp53 (Trp53em1Baek/J, PMID:27272387), ICR (OrientBio, Gyeonggi-do, Korea), severe immunodeficient mice (NOD/J-Prkdcem1BaekIl2rgem1Baek/J) (GEM Biosciences, Chenogju, Korea), Il17a (B6.129P2-Il17atm1Yiw, provided from Dr. Kyung Jin Lee from Asan Medical Center, Seoul, Korea), Meox2-cre (B6.129S4-Meox2tm1(cre)Sor/J, JAX, 003755), Vav1-iCre (B6N.Cg-Commd10Tg(Vav1-icre)A2Kio/J, JAX, 018968), Cdh5-cre (B6;129-Tg(Cdh5-cre)1Spe/J, JAX, 017968), R26R reporter (B6.129S4-Gt(ROSA)26Sortm1Sor/J, JAX, 003474), Tie2-cre (B6.Cg-Tg(Tek-cre)1Ywa/J, JAX, 008863)<br>7-week-old female mice were used for mating experiments. 6- to 8-week-old severe immunodeficient male mice were used for TS engraftment assay. 4-month-old Vav1-iCre mediated Pibf1 cKO male mice were used for blood cell count and hematopoietic potential assay.<br>All mice were maintained in the specific pathogen-free facility of the Laboratory of Animal Research at Asan Medical Center (AMCLAR)) at the ambient temperature of 23±1ºC and 50±5% humidity, with ad libitum access to standard laboratory food and water and a 12-h dark/light cycle.
Wild animals	We did not use wild animals.
Reporting on sex	The animals we used in this study were mostly conceptus samples comprising embryos and plaecnta derived from pregnant females. The male mice in this study were used as a stud for obtaining samples with interested genotypes after mating with females. The 4-week or 8-week-old pups was devided into male and female groups for their body weight measurement.
Field-collected samples	We did not use field-collected samples.
Ethics oversight	Experimental procedures were reviewed and approved by the Institutional Animal Care and Use Committees (IACUC) of Asan Institute for Life Sciences (approval number: 2019-12-217).

Note that full information on the approval of the study protocol must also be provided in the manuscript.

Plants

Seed stocks	n/a
Novel plant genotypes	n/a
Authentication	n/a

Flow Cytometry

Plots

Confirm that:

☒ The axis labels state the marker and fluorochrome used (e.g. CD4-FITC).

☒ The axis scales are clearly visible. Include numbers along axes only for bottom left plot of group (a 'group' is an analysis of identical markers).

☒ All plots are contour plots with outliers or pseudocolor plots.

☒ A numerical value for number of cells or percentage (with statistics) is provided.

Methodology

Sample preparation	Samples were chopped and digested using 1 mg/ml collagenase type II and 1 U/ml DNase I at 37 ºC for 30 min. The prepared single cells were Fc-blocked with anti-CD16/CD32 (553142, BD Biosciences) and then stained with antibodies.
Instrument	Stained samples were assessed using FACSCanto II system (BD Biosciences).
Software	Data were analyzed using FlowJo software (Tree Star).
Cell population abundance	In a single sample of decidua at E8.5 or E10.5 of pregnancy, 200-300 events on average were acquired in the defined gate.

Gating strategy

Gating strategies are provided in the supplementary figure 8.

☒ Tick this box to confirm that a figure exemplifying the gating strategy is provided in the Supplementary Information.
